# Evaluating Metabolic Profiling of Human Milk Using Biocrates MxP^®^ QUANT 500 Assay

**DOI:** 10.3390/metabo15010014

**Published:** 2025-01-03

**Authors:** Daniela Hampel, Setareh Shahab-Ferdows, Gilberto Kac, Lindsay H. Allen

**Affiliations:** 1 Department of Nutrition, University of California, Davis, CA 95616, USA; lindsay.allen@usda.gov; 2 United States Department of Agriculture, Agricultural Research Service—Western Human Nutrition Research Center, Davis, CA 95616, USA; setti.shahab-ferdows@usda.gov; 3 Nutrition Institute, Federal University of Rio de Janeiro, Rio de Janeiro 21941-902, Brazil; gilberto.kac@gmail.com

**Keywords:** human milk, colostrum, targeted metabolites, lipids, small molecules, LC-MS, FIA

## Abstract

**Background/Objectives:** Metabolic profiling of human milk (HM) is indispensable for elucidating mother-milk-infant relationships. **Methods:** We evaluated the Biocrates MxP^®^ Quant 500 assay for HM-targeted metabolomics (106 small molecules, 524 lipids) and analyzed in a feasibility test HM from apparently healthy Brazilian mothers (A: 2–8, B: 28–50, C: 88–119 days postpartum, n_total_ = 25). **Results:** Of the 630 possible signatures detectable with this assay, 506 were above the limits of detection in an HM-pool (10 µL) used for assay evaluation, 12 of them above the upper limit of quantitation. Analyzing five different HM-pool volumes (2–20 µL) revealed acceptable linearity for 458 metabolites. Intraday accuracy of 80–120% was attained by 469 metabolites after spiking and for 342 after a 1:2 dilution. Analyzing HM from Brazilian mothers revealed significantly lower concentrations in colostrum vs. mature milk for many flow-injection analyses (FIA) and only a few LC-MS metabolites, including triglycerides, sphingomyelins, and phosphatidylcholines. Higher concentrations at the later lactation stages were found predominantly for amino acids and related compounds. **Conclusions:** The MxP Quant^®^ 500 assay is a useful tool for HM metabolic profiling, minimizing analytical bias between matrices, and enhancing our ability to study milk as a biological system.

## 1. Introduction

Human milk (HM) is recognized as the optimal food source for infants [1] and recommended to be exclusively fed for the first 6 months of life while remaining an important dietary resource beyond [2]. Its complexity, compositional dynamic changes, and adaptation throughout lactation ensure that all physiological needs of the rapidly growing infant are met, emphasizing its significance to infant nutrition, growth, development, and disease protection [3,4,5,6]. Mapping the HM metabolic fingerprint has emerged as a vital tool for further insight into the variations in milk composition associated with maternal characteristics, such as health, diet, and environmental exposures, and the subsequent consequences for the infant.

While we do not fully understand how milk supports and influences infants [7], their long-term outcomes are shaped by the nutrients and substances transferred into HM through the maternal-milk-infant triangle [8]. Correlations have been reported between the HM metabolome and maternal diet, phenotype, and pregnancy-related complications such as pre-eclampsia or gestational diabetes [9,10,11,12], and there are differences between preterm and term milk metabolomes [13]. Human milk consumption in infancy has been linked to lower risks of inflammatory bowel disease, type 1 diabetes, and overweight in adulthood, and improved neurological outcomes, particularly in preterm infants [14,15].

Li et al. observed significant differences in the milk metabolome between milk from mothers of infants displaying typical vs. delayed neurodevelopment and based on infant sex. Some of the milk metabolites that were present in higher concentrations in the infants at risk group, e.g., acylcarnitines and phospholipids, are typical signatures of cell danger response (CDR) [16], indicating a customized metabolic maternal response through the milk to the infant’s stress [8]. We observed a comparable CDR with increased acylcarnitines and phospholipids in milk from Bangladeshi mothers of stunted infants [7].

Conversely, maternal physiological distress triggered changes in the HM metabolome, particularly increasing amounts of short-chain fatty acids [17]. Zhang et al. identified two HM metabotypes associated with slower infant growth, higher maternal body mass index (BMI), and increased risk of infant allergies [3]. Thus, the literature suggests a bilateral communication between mothers and their infants with HM as the mediator. To gain better insight, identical analytics for metabolic profiling of maternal and infant plasma and HM are ideal to produce comparable and robust results across different matrices without analytical biases.

We previously reported the validation and application of the Absolute*IDQ*^®^ p180 targeted metabolomics assay (biocrates life sciences AG, Innsbruck, Austria) for human milk, showing its usefulness after few adaptations from the original assay [7], and presented preliminary results in a conference abstract [18]. The purpose here was the full evaluation and application of Biocrates’ comprehensive MxP^®^ QUANT 500 assay for HM, covering up to 630 metabolites from 26 biochemical classes, including an extensive portfolio of triglycerides, the predominant lipid in HM. This assay has been validated for several matrices, such as plasma, urine, or feces, and while recently this assay was used for HM analyses, no detailed validation data was provided [19]. Hence, this is the first reported assay-evaluation using HM using this commercially available kit. For proof of concept, we used the assay for metabolic profiling of 25 milk samples from apparently healthy Brazilian mothers, collected between 2–8, 28–50, and 88–119 days postpartum, and examined longitudinal changes in the metabolic pattern.

## 2. Materials and Methods

### 2.1. Chemicals and Materials

Chemicals and materials such as reagents and test mixes, internal and calibration standards, quality controls, or the patented 96-well filter plates for the MxP^®^ QUANT 500 assay were purchased from or kindly supplied by Biocrates Life Sciences AG (Innsbruck, Austria).

### 2.2. MxP^®^ QUANT 500 Assay and Sample Preparation

The MxP^®^ QUANT 500 kit is a fully automated high-throughput assay employing a patented 96-well plate format covering up to 630 metabolites from 26 biochemical classes (13 small molecule and 13 lipid classes, Supplemental Table S1; https://biocrates.com/mxp-quant-500-kit/ [accessed on 20 November 2024]). Briefly, 10 µL of a whole milk sample is subjected to phenylisothiocyanate (PITC) derivatization, followed by extraction and dilution prior to analysis as per the manufacturer’s protocols, which are provided with each kit. In addition to the samples, a blank, zero sample, calibrators, and quality control are analyzed on each plate. Sample acquisition employs flow injection analyses (FIA) and liquid chromatography (LC) methods (Table 1). The raw data can be directly imported into Biocrates’ proprietary software (MetIDQ Oxygen was used for this study) for automated metabolite identification and quantification based on a simple target normalization procedure [20]. Details about the methodology, procedures, and instrumentation this commercial assay is based on are described in detail by patents EP1897014B1 and EP1875401B1 [21,22]. Supplemental Table S1 provides the full list of metabolites and their abbreviations.

### 2.3. LC-MS Systems

The LC-MS/MS consisted of an ACQUITY UPLC system (Waters, Milford, MA, USA) coupled to a QTRAP 5500 mass spectrometer (Sciex, Redwood City, CA, USA) in electrospray ionization (ESI) in positive and negative mode and multiple reaction monitoring (MRM) detection mode. Two acquisitions in each mode (FIA and LC-MS [LC-1 and LC-2]) were used for data collection. The FIA analyses were also carried out using a Waters Xevo TQ-S mass spectrometer for Biocrates in-house comparison purposes. Unfortunately, some FIA acquisitions using the QTRAP 5500 system were cut short due to instrument failure; hence, data acquired using the Xevo TQ-S system were used for FIA inter-day assessment and FIA dilution comparison (1:20 vs. 1:50 dilution. The results and conclusions were deemed transmissible given that the data obtained with both systems were of equally good quality (direct communications with Biocrates). Injection volumes ranged from 2 to 20 µL. LC-MS analyses were carried out using the MxP^®^ QUANT 500 kit column system, which is included in the kit and consists of a connector, pre-column mixer, and the analytical column. A 0.2% formic acid solution in water (solvent A) and acetonitrile (solvent B) were used in two different 5.8 min gradients to achieve the needed chromatographic separation (Table 2). FIA was performed employing a 3 min injection method; the FIA solvent was prepared from the FIA mobile phase additive provided by Biocrates and methanol (Table 2). All metabolites were identified and quantified using calibration standards and/or isotopically-labeled internal standards, employing MS parameters and multiple reaction monitoring (MRM) detection mode as evaluated and optimized by Biocrates Life Sciences AG (Innsbruck, Austria).

### 2.4. Human Milk Samples

A human milk pool (HMP) was kindly provided by an apparently healthy mother in the Sacramento, CA, USA area. Further, 25 milk samples from apparently healthy Brazilian mothers (individual milk samples—IMS), collected between 2–8 (group A), 28–50 (group B), and 88–119 days postpartum (group C), n_total_ = 25, were analyzed in a feasibility study. Five mothers provided milk samples at each time point; another five only had samples for 2 time points, for a total of 25 milk samples. All HM samples for this evaluation were shipped in dry ice from Brazil to the United States Department of Agriculture, Agricultural Research Service-Western Human Nutrition Research Center (USDA, ARS-WHNRC), Davis, CA, USA, on dry ice for other analyses, and then from the USDA, ARS-WHNRC to Biocrates Life Science AG in Innsbruck, Austria. Upon arrival, samples were stored at −80 °C until analysis in both laboratories.

### 2.5. Assay Evaluation

All pre-analytical and analytical procedures were performed, documented, and reviewed according to Biocrates’ ISO 9001:2015 [23] certified in-house quality management rules and guidelines.

#### 2.5.1. Concentration and Linearity

The analysis of the 25 IMS was carried out using 5 and 10 µL of each sample; 5 different sample volumes (2, 5, 10, 15, 20 µL) were used for HMP. Linearity and reproducibility were assessed using HMP by evaluating the coefficient of variation (CV) from the concentrations obtained from each of the 5 different sample volumes (CVs < 20% were acceptable, n = 2 for each sample volume). In addition, the FIA measurements were conducted in 1:20 and 1:50 sample dilutions, and the median sample concentrations in the LC measurements were compared to the highest and lowest levels of the calibration curve.

#### 2.5.2. Intra- and Inter-Day Variation and Analyte Recovery

Intraday variation was evaluated using the neat, diluted (1:2, 1:5), and spiked HMP (spiked using QC1, QC2, and QC3 plasma samples; n = 6 for each). Metabolite concentration had to be above the limit of detection (LOD) for at least 4 of the 6 replicates to be included in the evaluation. Intraday variations below 20% were acceptable. Recoveries of 80–120% were preferred, but recoveries outside this range with variation below 20% were acceptable. Inter-day variation was assessed by analysis of the 25 IMS on two different days by calculating the ratio of day 1 and day 2 measurements; accuracies between 70–130% were considered good, ranges of 50–70% and 130–200% were considered sub-optimal, while values <50% and >200% were unacceptable.

#### 2.5.3. Carry-Over and IS-Selectivity

Carryover was assessed by measuring phosphate-buffered saline (PBS; post-blank) after analyzing the HMP (each, n = 6) spiked with the high metabolite concentrations QC3. The signals obtained from these post-blank samples were then compared to a clean PBS sample set (n = 6, clean-blank). In cases where the post-blanks showed higher concentrations than the clean-blanks, carry-over was evaluated by comparing metabolite concentrations in post-analysis PBS samples to HMP samples. Values below 10% were acceptable.

Potential effects of the milk matrix on the internal standards (IS) were tested by examining the absolute quantification and assessing the selectivity of the IS-MRMs. The 25 IMS, HMP, and blanks were processed with and without IS, and the ratio of the IS-MRM intensities in the milk samples without and with IS was calculated. Matrix effects were present when ratio values were >5%. Selectivity was then calculated as the median of analyte concentrations in HMP without IS divided by the median of analyte concentrations in HMP with IS. Values between 70–130% were acceptable.

### 2.6. Data and Statistical Analysis

Raw data was processed at Biocrates Life Sciences in Innsbruck, Austria, using their proprietary Met*IDQ*^TM^ software version Oxygen. Descriptive statistics (e.g., mean, standard deviation [SD], coefficient of variation [CV]) were carried out in Excel 2016 and Excel 365 (Microsoft Corporation, Redmond, WA, USA). Mean was used for technical replicates of the same sample (e.g., HMP replicates), while median was used for summary data of the 25 IMS. Statistical analysis was conducted with RStudio version 2024-09-0 (Posit PBC, Boston, MA, USA) in conjunction with R 4.4.1 (R Foundation, Vienna, Austria). Differences in metabolite concentrations by time point (groups A, B, and C) were examined by pairwise comparison employing the Wilcoxon signed rank test to account for the repeated measures design. *p*-values < 0.05 indicated significance.

## 3. Results

### 3.1. Assay Evaluation

#### 3.1.1. Concentrations and Linearity

The usually used LC-MS acquisition method was modified to account for differences in metabolite concentration between the plasma QC and HM samples. Collision energies were optimized for lowabundant milk metabolites, such as polar analytes, and de-optimized for metabolites in saturation, e.g., the IS for phenylalanine, some amino acids (e.g., proline, lysine, glutamine, and taurine), and fatty acids (12:0, 18:0, 18:1, 18:2). Taurine and glutamate milk concentrations required higher calibration levels, expanding the standard 7-point calibration to an 8-point and 9-point curve. Other metabolites such as ADMA, dopamine, kynurenine, methionine, methionine sulfoxide, ornithine, and sarcosine were present in concentrations below the Cal-1 standard or below the lower limit of quantitation (LLOQ). Using only 5 µL of HM for analysis reduced the number of detectable metabolites without reducing signal suppression.

The FIA measurements were conducted with the standard dilution of 1:50 and a 1:20 dilution. While metabolites in the 1:20 dilution were not impacted by saturation effects, no advantages were found either. Unlike in the LC-MS analyses, instrument parameters for analyses did not need adjustment; only a milk-specific MetIDQ operating procedure was required to overcome selectivity concerns described below.

Linearity was assessed only for metabolites detected above LOD. Of the 79 detectable LC-MS metabolites (CV < 20%), 70 were linear, and 427 of 466 detectable FIA metabolites (CV < 30%) were linear (Table S2). Non-linear metabolites close to or above ULOQ included fatty acids FA(12:0), FA(14:0), FA(18:1), FA(18:2), FA(20:2), cysteine and cystine, lysine, as well as some di- and triglycerides (Table S2). Non-linearity was also observed for several low-abundant metabolites, close to LLOQ and LOD, e.g., trigonelline, some acylcarnitines, and phosphatidylcholines. Non-linear metabolites with concentrations above LLOQ and below ULOQ included selected di- and triglycerides, C14:2-OH, and Cer(d18:2/14:0); however, these metabolites (n = 23) reached linearity when excluding the two lowest sample volumes (2 and 5 µL; Table S2).

#### 3.1.2. Intra- and Inter-Day Variation and Analyte Recovery

Overall, satisfactory intraday variation (CV < 20%) was observed for 426 of 471 detectable metabolites. Only TG (18:0_38:7), Cer (d18:1/26:1), PC aa C38:1, and PC ae C38:2 had CVs > 30% (Table S3).

Good inter-day variation was found for 346 of 548 detectable metabolites when only considering metabolites with good accuracy in at least 15 of 25 samples (>60%; Table S3). Metabolites found in the sub-optimal inter-day variation category for most of the samples included AconAcid, FA(14:0), FA(20:3), and several lysoPCs, di- and triglycerides. Unacceptable inter-day variations in at least 10 samples were found for one LC-MS- (GABA) and 14 FIA metabolites, predominantly DGs (n = 8) and lysoPCs (n = 5), and one ceramide. The mean-to-median ratio of the inter-day variation across all metabolites ranged between 0.92 and 6.75 (mean 1.10, CV: 262%), but only three acylcarnitines had elevated mean-to-median ratios >2, namely C3-OH, C5:1, and C14:2-OH (Table S3).

Metabolite recovery after sample dilution (1:5 and 1:2) revealed 202 detectable 371 metabolites in the preferred recovery range (80–120%). When only considering the 1:2 dilution, this was true for 342 of 433 metabolites. AABA, Cys, FA (20:2), C14, and DG (14:0_14:0) were among the metabolites with elevated recoveries (>120%) but with good precision (CV < 20%). Fifty-five metabolites, including anserine, beta-Ala, PC ae C38:3, and TG (18:1_38:7), were detectable in both dilutions with elevated CVs, which fell below the cut-off only in the 1:2 dilution. Eleven compounds, among them GCA, spermine, C5-OH (C3-DC-M), and Hex3Cer(d18:1/16:0), were only detectable in the less diluted samples, but with higher CVs (Table S3).

**Figure 1:**
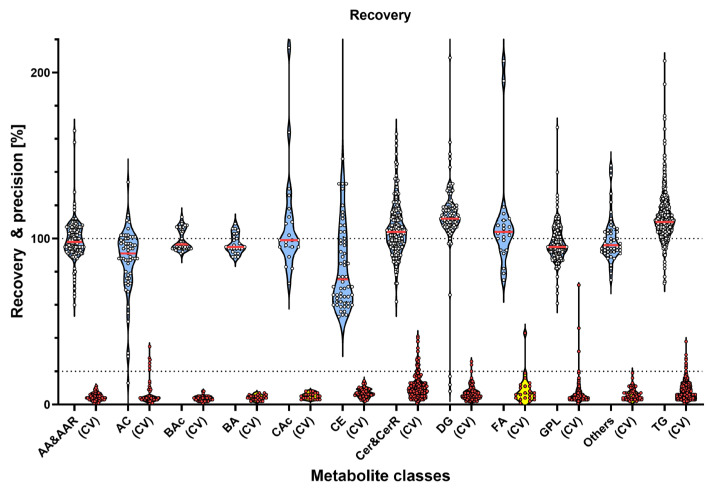
Violin plots of mean HM metabolite recovery (blue with yellow datapoints) and coefficients of variations (CVs; yellow with red datapoints) determined using a 3-level plasma-QC spike (n = 6). Metabolite classes are grouped as follows: AA&AAR—amino acids and related; AC—acylcarnitines; BAc—bile acids; BA—biogenic amines; CAc—carboxylic acids; CE—cholesterol esters; Cer&CerR—ceramides and related; DG—diacylglycerols; FA—fatty acids; GPL—glycerophospholipids; TG—triglycerides; Others—alkaloids, amine oxides, cresols, hormones, indole derivatives, nucleobases andrelated vitamins, and sugar.

Spike recoveries in the preferred range were observed for 470 of 567 detectable metabolites, of which 14 had CVs > 20%. Thirty-two metabolites were recovered <80%, of which five also had greater CVs. Sixty-five metabolites were observed with recoveries >120%, of which eight also showed CVs > 20% (Figure 1, Table S3). Orn was the metabolite with the highest overall spike recovery, over 700%, with a CV of 125%, but with good recoveries (89% and 91%) and reproducibility (CVs: 11 and 5%) in the 1:2 dilution and the QC1 spike, respectively. Similarly, DG (18:1_20:2) had high recovery rates with high variability (271.9%, CV 59.3%), but when examining each spike level separately, different recovery rates were found for each (151–455%) but with good reproducibility (4–7%). The same trend was found for CE (20:1) and to a lesser degree for CE (22:2).

#### 3.1.3. Carry-Over, Selectivity, Matrix Effects

Carry-over effects were observed for 26 metabolites (1 LC-MS, 25 FIA). Many were present in HMP below or close to LOD (LOD < 3× HMP concentrations). Only Cer(d18:/18:1), DG(18:3_20:2), and lysoPC a C26:0 were critical with carryover above 10% of the HMP.

Matrix effects were found for several LC-MS metabolites. Signals for anserine, sarcosine, spermine, spermidine, and putrescine were enhanced when the IS was present, while cystine revealed signal suppression under the same conditions. The IS used for taurine showed high intensities, even when no IS was present in the sample (Table S4).

Matrix-caused IS-signal suppression (10–25%) was observed for selected acylcarnitines (C3, C3-DC (C4-OH), C3-OH, C3:1, C4, C4:1, C6 (C4:1-DC), C6:1; Table S4). Similar to taurine, IS signals were detected in samples with no IS added for d3-C4, d3-C6, and d3-C16, and only sometimes for d9-C5. A severe intensity issue was found for the d3-C16 IS with a 40% response in samples without IS addition. PC ae 32:1 and PC ae C38:2 were the only FIA metabolites revealing a higher signal in the presence of their IS, while lower responses were observed for PC aa 32:1 and TG (20:5_36:2; Table S4).

### 3.2. Individual Human Milk Samples—Feasibility Plate

Analyzing the 25 IMS collected at three different stages of lactation revealed that 447 metabolites (LC-MS: n = 53, FIA: n = 394) were present in all samples above LOD regardless of lactation stage, while this was true for 510 metabolites (LC-MS: n = 64, FIA: n = 446) when including metabolites detectable above LOD in at least 60% (n = 15) of the samples (Table S5). Some metabolites, such as TrpBetaine, C4:1, CE22:5, PC aa C40:2, or PC ae C36:0, decreased over time to concentrations below quantitation or detection limits in some of the samples. LC-MS (n = 23) and FIA (n = 29) metabolites were below LOD in all 25 samples, among them several bile acids and about half of the acylcarnitines. All sphingomyelins, amino acids, and all but 6 of the 242 triglycerides were detected in all the samples. Unsaturated fatty acids FA18:1, FA18:2, FA20:1, and FA20:2 revealed levels >ULOQ but inconsistently (10–24 of 25 samples; Table S5).

**Figure 2:**
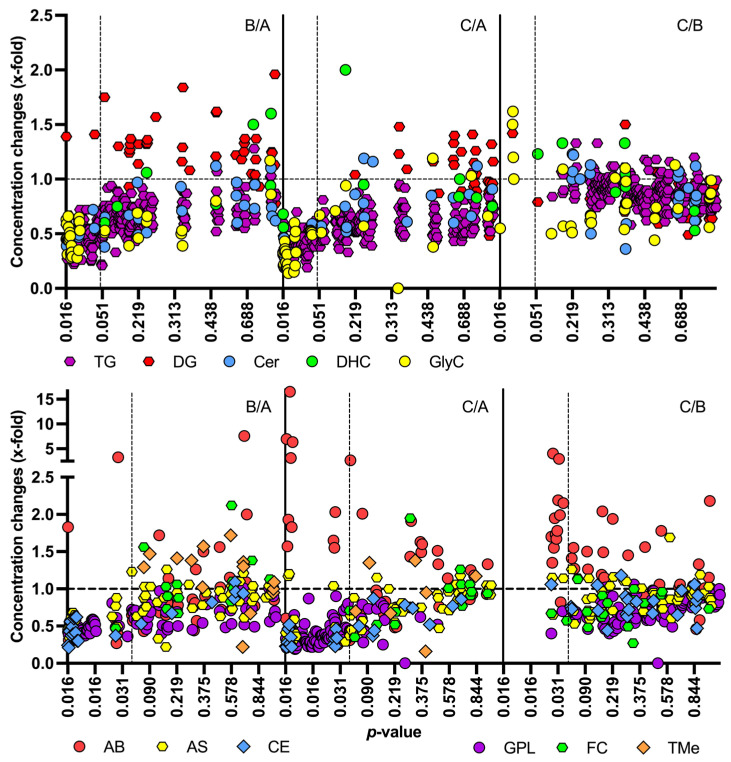
Median concentration changes (x-fold) of metabolite classes in 25 IMS from Brazilian mothers, separated by group comparisons [A—2–8 d, (n = 9); B—28–50 d, (n = 8); C—88–119 days postpartum (n = 8)]. B/A—changes from A to B, C/A—from A to C, C/B—from B to C. X-fold > 1—increasing concentrations; x-fold < 1—decreasing concentrations. *p*-values < 0.05 were considered significant (indicated by dashed vertical lines; Wilcoxon signed-rank test). Only metabolites for which concentrations and *p*-values were available are included. IMS—individual milk samples (n = 25), TG—triglycerides, DG—diglycerides, Cer—ceramides, DHC—dihydroceramides, GlyC—glycosylceramides (hexosylceramides, di- and tri-hexosylceramides), AB—amino acids and related biogenic amines, AS—acylcarnitines, sphingomyelins, CE—cholesterol esters, GLP—glycerophospholipids [(lyso) phosphatidylcholines], FC—fatty and carboxylic acids, TMe—trace metabolites [alkaloids, amine oxides, bile acids, cresols, indoles, carbohydrates-classes with only 1 or 2 detectable metabolites].

Four of the 13 LC-MS metabolite classes contained analytes that significantly changed in concentration over the course of lactation, while this was true for 12 of 13 FIA metabolite classes (Table 3 and Table S5): higher concentrations at later stages of lactation were found for most of the amino acids and amino acid-related compounds and for acylcarnitines (Figure 2). Most of the FIA metabolites decreased in concentration, e.g., all the triglycerides, ceramides, and phosphatidylcholines. Concentrations of choline, taurine, glutamic acid, FA (18:1), and FA(18:2) were above the upper limit of quantitation (ULOQ) in at least 23 of 25 samples (Table S5). Only four FIA metabolites were found >ULOQ, but only in 3 to 7 samples.

## 4. Discussion

### 4.1. Assay Evaluation

#### 4.1.1. Concentrations and Linearity

The complex matrix of HM presents challenges for any analytical methods, and its metabolite concentrations can be vastly different from those of other matrices such as plasma or serum. Here, adaptations to the plasma-optimized acquisition method and extension of the calibrator range were required to optimize the number of analyzable HM-metabolites, similar to our previous report using the Absolue*IDQ*^®^ p180 kit [7]. While matrix-specific interferences were expected, tests with different sample volumes and dilutions were not beneficial and resulted in fewer numbers of detectable metabolites. Therefore, the typically used sample volume (10 µL) and FIA dilution (1:50) remained superior and are recommended for this application. Further, high lactose concentrations in HM can impede the equipment and analysis [24]; this can be of particular importance to the FIA injections since the sample extract is directly injected into the MS, including all remaining matrix components. While the tested 1:20 dilution already reduces the lactose to less than 0.5% in the sample extract, the standard dilution of 1:50 even further reduces the presence of matrix, which is likely to aid in the higher number of observable FIA metabolites even at higher dilution by reducing matrix-induced signal suppression.

Our results indicated good linearity for about 90% of the detectable metabolites. Most of the non-linear LC-MS analytes were high-abundant compounds possibly in saturation, which may be resolved by a greater instrumental linear range or possibly by further de-optimizing their collision energy values. Non-linear metabolites were also found in the linear concentration range. For 23 metabolites, however, linearity was restored only with the optimal 10 µL sample volume. This greater variation in the low sample volumes (2 and 5 µL) results further supports that these volumes are suboptimal for this analysis.

#### 4.1.2. Intra- and Inter-Day Variation and Recovery

Satisfactory intraday variation was found for ~95% of the detectable metabolites. Out of the 26 metabolites with a suboptimal intra-day variation, only 4 had a CV > 30% (up to 36.6%), which included a ceramide, phospholipids, and a TG. All of them were found in low abundance with concentrations close to LOD, indicated further by their absence in the 1:5 dilution, with very low concentrations of two of the metabolites at the 1:2 dilution.

Inter-day variation evaluated using the 25 IMS was overall acceptable in over 80% of the detectable metabolites. Suboptimal or unacceptable overall inter-day variation was often found for low-abundant metabolites not observed in the HMP, including all the lysoPCs detectable in the 25 individual samples. Mean and median inter-day variations (IV) were comparable for most of the metabolites; only two acylcarnitines, C3-OH and C5:1, had a noticeably higher mean vs. median IV (3.8 and 6.8-fold, respectively), indicating that these metabolite concentrations are skewed across the IMS, as few of them revealed concentrations on the second day of analysis deviating considerably from the first-day results. To overcome these challenges and for better comparability over time, this assay unusually employs external target values established for the QC samples to normalize the acquired data. However, this requires a QC that matches the concentration and matrix of the actual samples. Hence, normalizing the HM data using the plasma QC was not successful but could be a useful tool when an HM-QC is available.

Elevated CVs above 20% were observed for considerably more metabolites in the 1:5 than in the 1:2 dilution and the non-diluted samples, particularly for DGs and TGs. This further supports that the concentrations shifted below LOD and/or LLOQ when diluting the sample, following the trend observed when using lower sample volume for analysis, as expected.

Spiking the HMP was carried out using plasma-QC samples at three different levels due to the unavailability of neat standards for some metabolites. Therefore, it was expected that not all metabolites would yield results. Plasma and HM contain metabolites at different levels, which will subsequently affect detection. e.g., if a metabolite is highly abundant in milk but low in plasma, a spike will not necessarily be noticeable, while high metabolite concentrations in both matrices can push the signal outside the linear detection range. Despite these pitfalls, using the plasma-QC increased the number of detectable metabolites and showed that metabolites not natively present in the HMP were nonetheless measured accurately and reproducibly. This data is useful as these metabolites may be present in other HM samples.

Orn and Val were the only two abundant metabolites with extreme values overall for spike recovery and CV. However, when only considering the QC-1 level, Orn had a recovery of 91% (CV: 5.4%), implying that the other QC levels may not be suitable for HMP spiking, likely due to saturation effects. This was also true for all QC levels for Val, resulting in only one QC level providing analyzable signals but around the ULOQ. However, both metabolites were accurately and reproducibly analyzed in the non-diluted and diluted HMP; hence these observations are spike-specific and do not indicate analytical challenges within the linear range but fall into the data affected by spiking HM with plasma. Contrarily, spiking did not push concentrations into the linear detection range for several metabolites, particularly acylcarnitines and ceramides. Since these metabolites were not typically present in HM analyzed here, the missing evaluation data was not crucial, but it should be noted that no data is available if those metabolites are present in other HM sample sets.

#### 4.1.3. Carry-Over, Selectivity, Matrix Effects

Carryover was not a concern for the LC-MS or FIA acquisition. All metabolites affected by carry-over were low in abundance in HMP with concentrations close to or below LOD, which was true for most of these metabolites when analyzing the 25 IMS. Hence, carryover was not critical in our analysis, and it is possible that affected metabolites are not commonly present in milk.

While the matrix affected several LC-MS metabolites, none of these interferences pushed the metabolite signals outside their linear range for quantification. Further, these metabolites are all calibrated, which allows for accurate quantification. Some observations may stem from other sources, such as the extremely elevated signals for spermidine and spermine. Since the IS was not detectable in milk samples processed without IS, its signal does not affect the analyte signal and therefore is unlikely to cause selectivity issues. Contrarily, chromatographic issues cannot be excluded. However, their steady recovery rates of >90% (CVs: 3.4%, 5.2%) indicate good accuracy and precision for both metabolites. The high IS intensities found for taurine may be explained by the similar MRM transitions with a mass shift of only +2 between metabolite and IS and the high abundance of taurine in milk causing some crosstalk between MRM transitions, resulting in a false positive IS signal as observed in samples analyzed without IS.

Examining the FIA acquisition d3-C16-IS produced a signal even when not present in the milk sample, which was not found in zero or plasma samples. Hence, this effect is most likely specific to the HM matrix. Using an alternative IS (d3-C18) for the affected 11 acylcarnitines resolved the issue and resulted in the expected higher concentrations for these metabolites. This adjustment successfully resolved this HM-specific phenomenon but required further modification of the MetIDQ operating procedures.

Compared to plasma samples, human milk caused severe signal suppression in both FIA and LC-MS analyses, which can cause false negative results when signals vanish due to suppression. However, this only affected FA (12:0), which was found only in some samples. Nevertheless, it is noticeable that analytes with suboptimal accuracy were also affected by suppression, identifying the latter as an additional source of variance.

### 4.2. Human Milk Samples—Feasibility Plate

The importance of HM to infant nutrition, growth, and development has been globally recognized. Nonetheless, many aspects of how HM supports the infant and insights into the mother-milk-infant relationship remain unknown despite the recent surge of HM research [5,7,25]. Within our validation efforts, we included a small sample set to examine longitudinal changes in metabolite concentrations throughout the first 3 months of lactation. Several studies have investigated longitudinal changes in the HM metabolome [13,26,27,28,29]. Overall, while only limited information was found on metabolite absolute quantification, concentrations for amino acids and related metabolites across these studies were comparable to our results. Consistently across all these studies, glutamate and glutamine were found in higher concentrations later in lactation, which agrees with our results. While most of the dynamic amino acids increased in concentration, we found lysine concentrations peaked within the first week of lactation, which was also observed in milk from Irish and Californian mothers using ^1^H nuclear magnetic resonance spectroscopy (NMR) [27,28,29].

Andreas et al. (2015) described a comprehensive multi-platform approach for HM metabolic profiling during lactation, defining 710 metabolites [26], including lipid classes that are not typically included when using ^1^H-NMR but covered in the MxP^®^ QUANT 500 assay, e.g., di- and triglycerides, phosphocholines, and sphingomyelins. Their cohort included hindmilk samples from the UK, collected between 2–80 days postpartum. We found similar trends in our study, including decreasing concentrations for phosphocholines, sphingomyelins, and triglycerides over time. Contrarily, we did not see concentration changes for fatty acids, while the UK cohort had higher concentrations at later lactation stages. However, the heterogeneity of the fatty acid portfolios for each study may contribute to different observations.

Recently, Calvo-Lerma et al. (2024) reported the application of the very same assay analyzing milk from Spanish mothers collected at 1 month postpartum [19]. While no individual concentrations for the metabolites were reported, the median sum of metabolite classes was comparable, with triglycerides being the most abundant (15,474 µM in group B vs. 12,660 µM [19]). The top four most abundant metabolite classes were identical between studies (triglycerides, fatty- and amino acids, sugars). Further, the number of metabolites detected in the 26 classes was similar between these studies; triglycerides were the only class for which we found considerably more metabolites (n > 20). These findings and their similarities further reiterate the usefulness of the MxP^®^ QUANT 500 assay for human milk metabolic profiling.

When comparing the two mature milk groups (B and C), we found a few metabolites with significant changes, but dihexosylceramides and amino acids were the predominant metabolite classes with increasing concentrations, indicating that the HM profile continues to change during the later lactation stages. Dihexosylceramides are glycosphingolipids, found in the milk fat globular membrane [30]. They are essential for nervous system formation and brain development, cell function, the formation of lipid rafts in the cells of the innate immune system, and they bind to pathogens and mediate immune and inflammatory responses [31,32]. Free amino acids are needed for metabolic activities; yet, HM only provides up to 5% of the infant’s needs while the gap is covered by protein intake [33]. Van Sadelhoff et al. (2021) reported an association of specific free amino acids in HM with infant growth at 4–5 weeks postpartum. Further, their consistently reported sharp increases in glutamate and glutamine concentrations in HM, also found in our data, point towards a tight regulation of these free amino acids postpartum. Both findings are indicators of the importance of HM-free amino acids to the rapidly growing and developing infant [34,35]. Hence, both metabolite classes have reported significance in infant development, which aligns with our findings of increasing concentrations during lactation, even in mature milk.

While we were limited in our sample size within this validation project, we were able to confirm previously published findings and tentatively found new information regarding the dynamic changes of the HM metabolome, which could indicate significance to the rapidly growing infant.

## 5. Conclusions

To our knowledge, this is the first published data on using the Biocrates MxP^®^ QUANT 500 assay for targeted metabolic profiling of HM, describing linearity, recovery, intra- and inter-day variation, carry-over, and selectivity. Typically, a 10 µL sample volume is recommended for this analysis, which holds true for HM, allowing comprehensive metabolic profiling even if only very minimal sample volumes are available. Analyzing a small sample set using the newly adapted methodology confirmed results from other studies showing the usefulness and validity of using this assay for HM. Further, we also found some novel aspects of the dynamics of the HM metabolome, which warrant further investigation. Thus, this assay was successfully adapted for this sample matrix, indicating that the assay, while not originally developed for HM, can indeed be used for its analysis, permitting the use of the same commercial assay for blood/plasma and HM, which will aid in further elucidating the mother-milk-infant relationships without the analytical bias of using different approaches for analysis. Having the same assay available to many different matrices will be indispensable for large multicenter studies, such as the Mothers, Infants, and Lactation Quality (MILQ) study.

## Figures and Tables

**Table 1:** Metabolite classes included in the MxP^®^ QUANT 500 kit and their detection modes.

Metabolite Class	Abbreviation	n ^1^	Detection Mode ^2^
Acylcarnitines	AC	40	FIA
Alkaloids	AL	1	LC-MS
Amine oxides	AO	1	LC-MS
Amino acids	AA	20	LC-MS
Amino acid-related	AAR	30	LC-MS
Bile acids	BAc	14	LC-MS
Biogenic amines	BA	9	LC-MS
Carbohydrates and related	CHO	1	FIA
Carboxylic acids	CA	7	LC-MS
Ceramides	Cer	28	FIA
Cholesteryl esters	CE	22	FIA
Cresols	Cre	1	LC-MS
Diglycerides	DG	44	FIA
Dihexosyl-ceramides	DHxC	9	FIA
Dihydro ceramides	DHC	8	FIA
Fatty acids	FA	12	LC-MS
Hexosyl ceramides	HxC	19	FIA
Hormones and related	Her	4	LC-MS
Indoles and derivatives	Ind	4	LC-MS
Lyso phosphatidylcholines	LPC	14	FIA
Nucleobases and related	NB	1	LC-MS
Phosphatidylcholines	PC	76	FIA
Sphingomyelins	SM	15	FIA
Triglycerides	TG	242	FIA
Trihexosylceramides	Thanks	6	FIA
Vitamins and cofactors	Vit	1	LC-MS

^1^ n—total number of metabolites for each class. ^2^ FIA—flow injection analysis; LC-MS—liquid chromatography-mass spectrometry.

**Table 2:** UHPLC parameters ^1^.

ACQUITY UPLC	Time [min]	Flow Rate [mL/min]	Solvent A [%]
LC-1	0.00	0.8	100
	0.25	0.8	100
	1.50	0.8	88
	2.70	0.8	82.5
	4.00	0.8	50
	4.50	0.8	0
	4.70	1.0	0
	5.00	1.0	0
	5.10	0.8	100
	5.80	0.8	100
LC-2	Time [min]	Flow Rate [mL/min]	Solvent A [%]
	0.00	0.8	100
	0.25	0.8	100
	0.50	0.8	75
	2.00	0.8	50
	3.00	0.8	25
	3.50	0.8	0
	4.70	1.0	0
	5.00	1.0	0
	5.10	1.0	100
	5.80	0.8	100
FIA ^2^	0.0	0.03	Biocrates FIA solvent running buffer in isocratic mode
	1.6	0.03	
	2.4	0.20	
	2.8	0.20	
	3.0	0.03	

^1^ UHPLC—liquid chromatography-mass spectrometry; ^2^ FIA—flow injection analysis, conducted in positive and in negative mode.

**Table 3:** Biocrates MxP^®^ Quant 500 metabolite classes found in 25 human milk samples.

Metabolite Class	Mode	n ^1^	n_sig.diff_ ^2^	Conc. ↑	Conc. ↓
Alkaloids	LC-MS	1/1	0		
Amine oxides	LC-MS	1/1	0		
Amino acids	LC-MS	20/20	12	10	2
Amino acid-related	LC-MS	21/30	13	7	6
Bile acids	LC-MS	7/14	0		
Biogenic amines	LC-MS	6/9	1		1
Carboxylic acids	LC-MS	5/7	3		3
Cresols	LC-MS	1/1	0		
Fatty acids	LC-MS	10/12	0		
Hormones	LC-MS	0/4	0		
Indoles	LC-MS	2/4	0		
Nucleobases	LC-MS	0/2	0		
Vitamins	LC-MS	1/1	0		
Acylcarnitines	FIA	11/40	4	3	1
Carbohydrates	FIA	1/1	0		
Ceramides	FIA	25/28	11		11
Cholesteryl esters	FIA	13/22	1		1
Diglycerides	FIA	39/44	2	1	1
Dihexosylceramides	FIA	9/9	8 ^3^	2	7
Dihydroceramides	FIA	1/8	1		1
Hexosylceramides	FIA	19/19	18		18
Lyso phosphatidylcholines	FIA	9/14	6		6
Phosphatidylcholines	FIA	59/76	51		51
Sphingomyelins	FIA	15/15	12		12
Triglycerides	FIA	240/242	104		104
Trihexosylceramides	FIA	5/6	2		2

^1^ number of detectable metabolites in at least one sample over number of metabolites within class. ^2^ number of metabolites from ^1^ with significant concentration changes ^3^ Hex2Cer (d18:1/20:0) significantly decreases in concentration and then increases.

## Data Availability

Data are contained within the article and Supplementary Materials.

## Supplementary Materials

The following supporting information can be downloaded at: https://www.mdpi.com/article/10.3390/metabo15010014/s1, Table S1: Biocrates MxP^®^ QUANT metabolite information, Table S2: Linearity of human milk pool metabolite concentrations, Table S3: Summary data of intra- and inter-day variation and recovery after dilution and 3-level spiking of a pooled human milk sample (n = 6 for all); Table S4: Selectivity and carryover evaluation using the 25 individual samples and the HMP; Table S5: Median concentration (µM) and interquartile ranges (IQR) of 25 milk samples from Brazilian mothers, collected at 2–8, 28–50, and 88–119 days postpartum.
